# Changing Body Image and Well-Being: Following the Experience of Massive Weight Loss and Body Contouring Surgery

**DOI:** 10.3390/healthcare2020150

**Published:** 2014-03-27

**Authors:** Jo Gilmartin, Andrew F. Long, Mark Soldin

**Affiliations:** 1 School of Healthcare, Baines Wing, University of Leeds, Leeds, LS2 9UT, UK; E-Mail: a.f.long@leeds.ac.uk; 2 Department of Plastic Surgery, St. George’s Hospital, Blackshaw Road, Tooting Broadway, London, SW17 0QT, UK; E-Mail: marksoldin@gmail.com

**Keywords:** massive weight loss, body contouring surgery, changing body image, adjusting to the changing body, improved well-being

## Abstract

This paper reports on the perception of changing body image and well-being for patients who had undergone plastic surgery following massive weight loss. The exploratory, qualitative study was undertaken with 20 patients from one teaching hospital in the south of England. Semi-structured interviews were carried out and a thematic analysis of the data undertaken. The results provide important insights regarding body contouring influencing body image change and the adjustment process involved. The ability to pursue self-esteem and the accruing social benefits is emphasized in the interrelated sub themes including social acceptance, undoing depression and sexual vitality. Body contouring surgery following massive weight loss appears to facilitate improvement in body image and well-being. Adjustment to the changing body image is both empowering and challenging. Supportive educational programmes need to be developed to assist this transition to a more positive body image and appreciation; these could usefully include access to and involvement with patient support groups.

## 1. Introduction

The global obesity epidemic is a major public health challenge, raising the potential of early heart disease, the onset of diabetes and a greater risk of mortality. In the past 30 years, obesity has increased in most countries of the world [1]. Weight loss following bariatric surgery is in the short term associated with significant improvements in obesity related comorbidities [2], body satisfaction and psychosocial outcomes [3]. A recent meta-analysis compared bariatric surgery with non-surgical treatment of obesity, and indicated that bariatric surgery leads to greater weight loss, higher remission rates of diabetes and greater improvements in quality of life [4]. However, in the context of extreme weight loss, body image and appearance may again worsen because excess skin may form an abdominal overhang [5]. Typically, the loose ptotic skin envelopes and residual adiposities can cause hygiene problems, rashes and mobility challenges with negative repercussions on body image, psychological well-being and social acceptance [6]. Thus, a high proportion of such patients are motivated to pursue plastic surgery to improve function and well-being, and overcome appearance related distress [7]. To remove excess skin, a variety of different plastic surgery methods are employed, such as abdominoplasty/tummy tuck, inner thigh lift, breast lift, arm lift and back lift. These are commonly referred to as “body contouring” procedures [8].

First, however, it is crucial to explain how we use the terms massive weight loss and body image. Obesity grade 111 (morbid obesity) is defined by a body mass index (BMI) of 40 kg/m^2^ or greater, with or without comorbidities [2,5]. Massive weight loss is defined as 50% or more of excess weight [9]. The criteria for body contouring within the National Health Service (NHS) include a starting BMI above 40 kg/m^2^ or above 35 kg/m^2^ with co-morbidities and a current BMI of less than or equal to 27 kg/m^2^ and weight stability of 12 months [10]. This eligibility criterion is important for specifying health risk.

As a result of the dissatisfaction with body image and obesity, there has in recent years been a huge growth in research [6]. Body image is defined as “the perception of overall physical appearance” [11] (estimated size of the body) and other writers allude to its attitudinal component, focusing on satisfaction with, and evaluation of, the body [12]. It is considered to be a major component of global self-esteem. Moreover, body dissatisfaction, while a component of body image disturbance, consists of dysfunctional, negative beliefs about one’s weight and shape [13]. Both body image and body dissatisfaction are subjective concepts and potentially open to change over time through social influence and self-reassessment [11]. For example, whilst obese people usually drive their change in appearance through weight loss surgery, the resultant excess skin tends to be unwanted and can trigger appearance-related distress and anxiety.

Studies investigating patients’ experiences after weight loss surgery showed that surplus skin caused fungal infections and itching, and problems with appearance and physical activity, such as undressing in swimming pools [14,15,16]. These studies reported that the desire to reduce surplus skin was largely motivated by body dissatisfaction. More recent studies [16,17] showed that following massive weight loss patients reported problems with appearance, physical activity and finding clothes that fit appropriately, and desired reconstructive surgery. However, despite such studies reporting distress at the disfiguring effect of excess skin, the implications for body image are not thoroughly considered.

Most of the existing, but moderate body of evidence exploring the benefits of body contouring surgery following massive weight loss arises from quantitative studies. These report improvements in physical functioning and feelings of healthiness [5,6]; improvements in psychological and mental health well-being [18,19]; stability in mood [6]; body image satisfaction [19]; feelings of attractiveness [6]; reduced feelings of body uneasiness [20]; enhanced self-image and self-esteem [21], including confidence [6] and positive thinking [20]; improved social acceptance [6,19], and greater involvement in social and cultural activities [22,23]. However, none of these studies provides substantial insight into the complexity of factors participants might experience in a changing body image. This reinforces the need for qualitative research such as that undertaken in the study reported here, which aims to provide detailed insight into patients’ psychosocial experiences of adjusting to body image change. Against this background, the paper reports on findings from a small-scale exploratory and qualitative study, and centres on the perception of changing body image and well-being for patients who had undergone plastic surgery following massive weight loss.

## 2. Methods

The study adopted a retrospective focus, drawing on a sample of patients from one clinical location providing body contouring surgery following massive weight loss. The primary data collection method was in-depth interviews, undertaken in participants’ homes or a setting of their choice (for example, work environment after office hours). The intention was to gain insight into, and understanding of, their underlying values, experiences and norms, using face-to-face in-depth interviews [24] which encouraged the participants to take the lead and shape their own disclosures [25]. Additional data were also sought, with the participants’ permission, from the medical records at the clinical location. NHS Ethics and Research and Development committee approval was obtained for the study. In addition, the participants were allocated pseudo names in the text to uphold anonymity.

### 2.1. Sample Selection and Recruitment

The research team retrospectively selected patients who had undergone body contouring in the past five years at the Plastic Surgery Department in one teaching hospital in the south of England. The aim was to collect a diverse sample of 20 participants, including different age ranges (at least 18 years and beyond), diverse ethnicity and cultural backgrounds, and ideally representation from both women and men, recognizing the heavy preponderance of body contouring surgery in women [22,26,27]. For example, fifty female patients were recruited by Pecori *et al.* [22], twenty-seven patients (25 females and 2 males) were selected by Koller *et al.* [26] and thirty women and four men were included in the study undertaken by Stuerz *et al*. [27].

Patients who were not fluent or had difficulty in understanding and speaking English were excluded from the current study. Forty-two of the 47 patients (44 females; 3 males) on the plastic surgeon’s database met these criteria. Invitation letters and an information sheet were then sent out to each of the 42 potential participants (39 females; 3 males). Those willing to participate in the research were asked to contact the lead researcher (JG), who responded to telephone calls, letters and e-mails, providing more information about the study and collecting baseline information on demographic details and type of body contouring procedures. Twenty-two people expressed a willingness to participate. However, at the point of setting up the research interviews, two withdrew because of family crises, resulting in a sample of 20 white adult patients (females 18; males 2).

### 2.2. Data Collection

Twenty participants were interviewed once in a community setting. The researcher obtained written consent before proceeding with data collection. An interview guide with open-ended questions was used. Examples of interview items asked of each participant were: How did you feel about the excess skin? What was the trigger to undergo plastic surgery? How did you feel about your body after surgery? How do you feel when you see your body, right now? How has your body image affected your lifestyle? A number of prompts were also employed to encourage participants to explore issues in greater depth. For example, how do you experience your body now? Interviews were audio-taped and transcribed verbatim. The researcher also took notes throughout the interviews and logged reflective thoughts in a diary after the interview ended. The duration of the interview varied between 1–2½ hours.

Subsequent to the interview, with the participant’s permission, relevant clinical data were abstracted from the medical records by the plastic surgeon’s medical secretary. The researcher read these records retrospectively in the Plastic Surgery department and gathered demographic information and data on comorbidities, body mass index, eating behaviour and lifestyle, bariatric surgery uptake, and body contouring procedures. The data were recorded and stored on a password protected memory stick.

### 2.3. Data Analysis

Data were analysed thematically [28] using NVIVO software for qualitative data management (version 7). Having carried out a familiarisation process and noted initial ideas, the lead researcher (JG) analysed each interview to develop a thematic coding scheme. Codes were developed using both open and selective coding processes in a systematic fashion across the entire data set, collating data relevant to each code (for example, body shape and size, body image, increasing body esteem, social acceptance). Initially 115 codes were developed. Examples of the codes and selected content were then read by another researcher (AFL), confirming or otherwise the relevance of the codes for the data. The researchers discussed the codes and considered links between them, before agreeing a final theme and associated sub-themes. To ensure rigour, appropriate guidelines [29] for ensuring trustworthiness were employed. The opportunity to comment on their findings was given to the participants, two of whom read and confirmed the researchers’ interpretation.

## 3. Results

### 3.1. Participants

Eighteen were females and two males. The mean age was 46.2 years (range 29 to 63 years). Six participants underwent single type reconstructive surgery procedures such as abdominoplasty (tummy tuck) and fourteen had multi-stage procedures including abdominoplasty, inner thigh lift, breast lift and upper arm lift. The mean BMI pre-bariatric surgery (Laparoscopic adjustable gastric banding or Sleeve gastrectomy or Roux-en-Ygastric bypass) among the16 patients who chose obesity surgery was 51.11 kg/m^2^ (range 40–70 kg/m^2^) and the mean BMI pre-dieting and lifestyle changes among the remaining 4 patients was 48.73 kg/m^2^ (range 38.4–55.3 kg/m^2^). The mean BMI pre-body contouring was 25.9 kg/m^2^ (range 20–35 kg/m^2^) for the 20 participants and the interval between bariatric surgery or weight loss and reconstructive surgery was 2–5 years. Half of the participants had a history of depression (commonly due to childhood maltreatment) as recorded in the patient notes. This included systematic sexual abuse by a family member (40%). On-going improvements or a reduction in the occurrence of depression were reported following body contouring surgery. For example, 10 people with mobility problems reported no on-going symptoms after plastic surgery. Nine subjects pointed to considerable improvements in mood swings/depressive episodes but five people alluded to ongoing difficulties with depressive symptoms.

### 3.2. Changing Body Image

At this juncture, the intention is to consider one core theme that emerged, changing body image, and its related sub-themes (undoing depression, social acceptance and sexual vitality). Other findings included identity transformation and changed lifestyle following body contouring surgery [30,31]. Participants reported on such important features that influenced their changing body image in the post-surgical body, and all implied that the plastic surgery was remarkable in terms of enhancing body image, self-esteem and attractiveness. Nonetheless, the process appeared to be transitory. Sixteen participants referred to scar severity and its long lasting impact, describing it as a defining element of their appearance and self-perception. The salience of scar severity and individual values on the importance of appearance seemed to be influenced by subjective traits, such as scar visibility and reactions to the scarring. There was substantial similarity between participants within these themes, though differences were also presented in their accounts of early and late scarring.

### 3.3. The Core Theme: Changing Body Image

Early scarring accounts were expressed in highly negative ways within the first year following surgery. For example, ten women mentioned looking “mutilated”, “ugly”, “shocking”, and the impact of others’ reactions. Nicole, aged 29, explained:

“*I’m like Frankenstein. I’ve got a long scar that goes all the way down my abdominal area. Once, um, I was having a bra fitting and the lingerie assistant saw it and said* ‘*Oh my God! Have you been burnt’I didn’t want to be spoken to like that*”.

Prudence, aged 58, said:

“*The scarring is immense. I look like a rag doll underneath my clothes. I do not like people looking at my body*”*.*

Estelle, aged 53, recounted:

“*I had massive scarring and I still have lots of stretch marks. I’m never going to look good without clothes on. I will never look like a glamorous young woman*”*.*

As these extracts show, early scarring was troublesome, being perceived as a salient feature that impacted on body esteem and well-being.

In contrast, late scarring accounts were much more positive and enthusiastic, alluding to increased body esteem and dramatic shifts in appearance. The decline in salience was mainly attributed to the time (1–5 years) following body contouring procedures, suggesting that many had become accustomed to their new body image. For instance, several respondents described their scars as “*fading*” and “*shrinking*”, with others saying “*looking normal*” and expressing feelings of self-worth. Grace, aged 51, asserted:

“*The scarring was not really nice… at first, but now it’s a few years on, they just look like thin, faint lines. They’re shrinking away*”*.*

Whitney, aged 63, commented:

“*I feel very different about myself now, a significant impact, for the first time in years I actually feel more or less normal. I started to take huge pleasure in clothes. I’m recovering lost time*”*.*

Jewel, aged 37, disclosed:

“*I feel excellent about my body now…eutrophic. I feel really good about my appearance and my self-confidence had a massive boost. I like mirrors now & I’m all right looking at myself*”*.*

Sophie, aged 47, remarked:

“*I feel wonderful now…I’ve got scars and they are visible but I hide them with my underwear. They do not bother me. I feel fantastic about my new body image and sense of self-worth*”*.*

Late scarring identifiers differed from early scarring ones in that they expressed less concern about the scarring. They were trying to recover lost time, invest in wearing lovely clothes and celebrate their new sense of self, increasing body esteem and well-being.

### 3.4. Social Acceptance

The positive, creative shifts described in the changing body theme have connections with the powerful sub-theme of social acceptance. After the plastic surgery the participants saw themselves as being on a dynamic trajectory, being accepted and integrated into society, rather than stigmatised and downgraded. Success stories are avowed in the following excerpts.

Harriet, aged 62, said,

“*I feel suddenly more energised following all the operations. My changing body has put an end to all the* *‘couldn’t do’. I walk every day and I’ve just got a bicycle and love cycling in the park*”*.*

Fife, aged 42, observed:

“*I love dancing and I set up a market cafe. This has been a long term ambition and I really enjoy interacting with the customers. This has been a major leap forward*”*.*

Iona, aged 39, commented:

“*I went to Barbados on honeymoon and I did go on the beach in my new swimsuit and sarong and there were other people around. I would never have done that before. Mentally this was a huge change and a massive step forward. I wore beautiful ‘sun dresses’ and ‘halter neck dresses’ too*”*.*

These “success stories” suggest that plastic surgery helps to produce a dramatic, visible change in appearance, creating happiness and an improvement in well-being. They also point to the importance of external validation, seen as firmly lying in the social world.

The majority of participants seemed to carry internalised notions of desiring active participation in local communities and becoming highly dynamic and involved. This sentiment is expressed in the following extracts. Aaron, aged 44, stated:

“*I am more active in a local theatre group. I joined a writing group on line to connect with new people. I met my husband in the writing group. We are hoping to write some plays together*”*.*

Kate, aged 38, avowed:

“*I am very active now…I manage my cleaning business and have great clients. I enjoy outings with my family and take the children to theme parks & holidays in the Med. I could not fit in the theme park rides previously with all the ‘excess’, loose hanging skin*”*.*

The positive psychological effects that come with body contouring surgery enabled greater social engagement and acceptance. This is partially reflected in the motivating desire of the respondents to engage and participate socially.

Fourteen participants highlighted the fact that partners, friends and colleagues became accustomed to their new appearance and ceased commenting on it. This transition was reported to lessen the intensity of negative social feedback and served to help internalise self-acceptance, improve mood and validate body image. Felix, aged 56, explained:

“*The surgery was brilliant and I feel confident, sexy and fabulous. I guess my outlook towards my appearance is different…. I make more of an effort for it which gets me better rewards. I feel like a recycled teenage and life is marvellous*”*.*

### 3.5. Undoing Depression

The themes of changing body image and social acceptance are characterised as “undoing depression”, marked by positive mood shifts, alongside the tension of never feeling completely safe from the threat of childhood demons. Throughout the interviews a subgroup of ten participants disclosed shocking accounts of sexual, psychological or physical abuse that triggered disordered eating, severe body hatred and depressive states. For example, Guy, aged 55, told the researcher,

“*I suffered severe physical and psychological abuse in childhood. My father hit me persistently with a cricket bat and a chair…broken nose…, black/bluish bruising all over my body. I never felt loved by my family. I had huge problems with alcohol addiction and emotional eating as a teenager. I still take antidepressants*”*.*

Aaron also disclosed:

“*My childhood years were awful, my mother and grandmother had a boarding house and I was sexually abused by male lodgers. This was damaging, cruel and terrifying*”*.*

Although Aaron underwent long term psychodynamic therapy, the lingering marks of childhood abuse still came to the fore and interrupted rehabilitation and well-being.

Fourteen participants alluded to a new sense of body, enabling a slow translation into positive self-esteem, self-confidence and an increased sense of psychological well-being and energy levels. Several commented that the plastic surgery helped modify the relationship between body image concerns and depressive symptoms. Noticing shifts in body image was reported as a turning point and seemed to enable the participants to consider body image in a more positive way. Among those with success stories, most reported sustaining a positive mood without the on-going use of prescribed anti-depressants. For example, Grace, aged 51, asserted,

“*Of course your moods are better but I need counselling or access to a support group because you’ve gone from this big person that was 30 stone…plus, to someone who is now like 12 stone but your mind and self-perception are still exactly the same…quite hard to adapt emotionally to the changing body*”*.*

Jewel, 37, told the researcher,

“*I always envisaged that my mood and self-confidence would be better but I never visualised how much. I can’t comprehend the massive, positive impact this had. My alcoholic, abusive mother died recently. I went to the funeral but do not wish to see my Dad again. I’ve closed that chapter and need to move on and embrace the ‘new me’”.*

However, six respondents had self-perceived on-going depression and anxiety symptoms characterised by appearance fixing or underlying thought patterns about body image. Two talked about emotional turmoil—the heightened use of internal, depressive, dysfunctional thoughts coupled with a desire for social isolation. Indeed, the concerns were related to body dysmorphia, recounted clearly by Chloe, 44,

“*It’s taken me quite a few years to get used to the scarring. I used to be, still am a bit dysmorphic. That triggers depressive thoughts. Erm, I don’t think that will ever change*”.

This particular respondent also mentioned experiencing transient suicidal feelings that appeared to have lessened following sustained weight loss and the appearance change after surgery. Estelle, aged 53, recounted:

“*I was caring for Mum suffering from dementia following the tummy tuck & I had a nervous breakdown and was admitted to hospital for almost a year. I felt overwhelmed with the ‘excess body’, & ‘excess caring’. I was put on antidepressants and I had CBT group therapy & undertook art classes, yoga and joined a carer’s support group. This was a powerful healing experience & I still take antidepressants because my Mum died recently*”*.*

### 3.6. Sexual Vitality

This sub-theme is linked to growing confidence and an enhanced sexual well-being and activity following the new visible appearance. Since surgery, most claimed that their attitude towards their appearance had changed; this included the application of beauty products, jewellery and make up, grooming, exercising regularly, and taking interest in contemporary lingerie and designer fashion. For all the participants, sexual vitality proved to be another important turning point, benefiting well-being. The influence of the partner’s view of their visual appearance seemed to contribute to validating their post-weight loss bodies.

Sexual satisfaction during sexual experience appeared to be more enhanced too because, in this context, their bodies were available for viewing and linked to sexual pleasure. Several said that they felt like a “new sexual being” and energised; this translated into sexual esteem and increased sexual functioning. Sexual competence is portrayed in the following excerpts. Ruby, aged 46, confided:

“*Um, I’m a lot happier about sexual intimacy, in fact, I say happier, but my husband says that I basically ‘flaunt myself’ at him now; whereas before surgery, I couldn’t bear him seeing me without my clothes on*”*.*

Whitney, aged 63, explained:

“*Yeah, definitely, for the first time in years, I’m looking good, and attract more sexual attention from my husband and limitless sexual pleasure. I make more of an effort with my appearance now*”*.*

Since surgery three women reported getting married and talked about a new expansiveness and joy, which are tremendously appealing and fulfilling. Iona, aged 39, recounted:

“*I got married last year and my husband worships me. I feel happier and more relaxed. I thought I would stay single all my life because I do not have a ‘perfect body’”.*

Moreover, several of the single participants mentioned attracting more sexual attention and starting new relationships that brought delight and pleasure. They engaged with social networking or internet dating to facilitate rapport building and intimate connections. Although most were enthusiastic about relationship aspirations, a few felt nervous about intimacy with a new partner because of the scarring and lack of a perfect body. For example, Bianca 47, disclosed:

“*I’m self-conscious and I feel that I’ve got to blurt it out and tell him before…. I’ve got to say, oh, by the way, I’ve got this massive scar on my stomach. I’ve got this. I’ve got that. Um, so yeah, it’s affecting how I have a relationship*”*.*

Even in this extract, however, it is possible to catch a glimpse of the body’s vulnerability and the challenges individuals may encounter in navigating new intimate relationships.

Among the men, one participant reported relentless attention to his body in terms of grooming, exercising daily, using creams and beauty products. He recounted re-entering the “*singles market*” after his divorce and becoming aware of the very competitive aesthetic standards for men that were different from those of married men. He felt it was necessary to display a “very attractive” aesthetic in order to meet a special new partner. This man appeared to accept the responsibility of a continuous regimen of diet and exercise.

## 4. Discussion

This paper has centred attention on participants’ changing perceptions and experience of body image following body contouring surgery after massive weight loss. The results suggest that the experience is complex, the body undergoing significant and multiple shifts and appearing to be in continuous state of flux. Perceptions of body image were consequently highly diverse, ambivalent and shifting. Such findings suggest a more nuanced interpretation than that presented in previous research [21,23], which pointed to body image satisfaction within three to six months following body contouring surgery.

The study’s findings cast additional light on the nature of improved body image perceptions as measured through quantitative instruments such as the body image and satisfaction assessment instrument [5], and the body uneasiness test [22], commonly used in similar studies. The widespread reliance on such measures underplays potential variability, running the risk of giving a false or superficial picture.

Our participants reported considerable distress in relation to body image, including feelings of shame, disgust and ugliness, for the first year following surgery. For the majority, long and intense scars contributed towards their distress. The psychological perspective presented by Lazar *et al*. [20] also pointed out that, after lengthy suffering from obesity, the emerging body image can become a source of deep shame and humiliation. Our findings also cohere with the development and maintenance of shame in such a visible difference [32], where experience of stigmatisation can lead to a “self-schema”, associated with increased anxiety about social encounters, concealment and sensitivity to further rejections. Nonetheless, most participants in our study reported an improved body image, after the first year, and in particular less distress related to their considerable scarring, highlighted in previous quantitative studies [6,19].

The study findings demonstrate clearly that changing body image and appearance have implications for social acceptance, losses as well as gains being embedded in participants’ accounts. “Fat” is a deeply stigmatized status [33] in Western cultures; “thinner” body shapes are more akin to the cultural ideal and are prized by society. Evidence from a meta-analytic review [34] demonstrates that exposure to thin media images negatively affected body image, particularly in women, those with high levels of body image concern and those with high internalization of the thin body ideal [34]. Our findings indicated that when the participants were more attuned to body acceptance or made connections between positive self-evaluations and self-esteem, the attuned traits seemed to elicit others’ acceptance [35]. However, most massive weight loss patients have long histories of “excess bodies” that appear to trigger ongoing tensions between body esteem in a social and cultural context that reward smooth muscular bodies [36].

There is also striking evidence in our findings of the participants transcending depression and emerging into a new serenity. They vividly described moving away from feelings of shame and disgust towards their bodies to appreciating the experience of the body in the present (1–2 years post-surgery). This is further supported by the extensive efforts made by participants to “let go” of anti-depressant medication and begin to adjust to and accept their changing body image. Their evolving experiences provided something to celebrate, as a new “body” and “body image” unfolds. This corresponds with previous findings suggesting an improvement in mental health well-being following body contouring surgery [6,20]. However, a few of our respondents did not pass through their depression, but rather struggled with adjusting to the new body image. It is likely that both the depression and the recovery lag probably had their roots in either Body Dysmorphic Disorder (BDD) or childhood trauma. Interestingly, “obesity research” has inferred links between childhood abuse, psychiatric diagnosis and eating disorder in the population [37]. A higher prevalence of axis 1 psychiatric disorders, most commonly anxiety and mood disorders, is also suggested [37]. Nonetheless, these findings vary greatly. In addition, individuals with BDD diagnosis have been found to have high discrepancies between their “actual” and “ideal” self [38]. They are thus likely to experience dejection-related emotions such as sadness, disappointment or dissatisfaction, where one’s hopes and aspirations are unfulfilled [39].

Finally, our findings demonstrate that participants’ changing body image improved their sense of sexual well-being and enabled them to be more active and dynamic in intimate relationships or in attracting sexual attention, a finding that coheres with other studies [6,33]. In some instances, the accounts given relate to “personal” growth and transformation alongside body transformation, in the sense of developing behaviours that were not present before. This is most strongly demonstrated where participants reported that sexual vitality proved an important turning point, translating into sexual esteem and increased sexuality functioning. However, some participants, despite having changed considerably in body shape and size, still seemed to be experiencing shame and anxiety, especially in revealing their naked bodies in a new relationship. This physical unease at being looked at by sexually interested individuals might trigger defensive avoidance behaviour, in situations that might cause emotional or even stronger reactions. As asserted in the literature [40], some individuals have a stronger coping capacity to tolerate all emotions without acting out defensive behaviours, whilst others find shame intolerable and go to great lengths to avoid situations that might induce it.

### 4.1. Implications for Health Professionals

Findings from the analysis suggest that both internal and external shame play a significant part in maintaining the negative experiences of the participants. Moreover many appear to be struggling towards self-acceptance and tend to focus on the “past”, rather than the here-and-now. Therefore, health professionals will face considerable challenges when they encounter service users with intense scarring following body contouring surgery. In an attempt to empower clients to achieve their individual outcomes in terms of body image enhancement, support and education are likely to be extremely important. It may be useful for healthcare professionals to facilitate body image acceptance programmes [41,42,43,44], and/or offer involvement in support groups. The support groups might enable participants to “notice blocks” and “let go of self-criticism” before engaging with body image acceptance programmes. In addition, the employment of mindfulness cognitive behavioural therapy strategies [45] might also be useful to facilitate positive effect “positive adjusters”. Concepts of “compassion” and “acceptance” are increasingly central in the third wave CBT models [46], acceptance and commitment therapy [47] and Gilbert’s compassion mind work [45]. These therapies appeared to be effective when working with individuals who are struggling to adjust to body image or appearance related concerns [48,49]. In the United Kingdom and globally, the management of massive weight loss patients who undergo body contouring surgery is facilitated mainly by specialist practitioners, whose expertise is in great demand. There is a need to involve the whole multi-disciplinary team, in both weight loss and body contouring surgery interventions, to add in and/or develop supportive rehabilitation programmes that address changing body image concerns. Therapeutic groups might be a useful intervention here. In addition, cognitive behaviour interventions have been found to be effective in addressing body image dysphoria, depression and anxiety [50].

### 4.2. Study Limitations

One notable limitation of the study is its small scale (n = 20), undertaken with participants drawn from a single surgical centre. Such a study size is appropriate for an exploratory study aiming for rich and informative data on people’s experience [51]. It was also limited by the focus on only one surgical centre and its patient group. In addition, the study adopted a retrospective view, recruiting participants 2–5 years post-body contouring and who were also unintentionally a more homogeneous ethnic group. Although such limitations must be borne in mind, the findings are nevertheless congruent with existing research. Moreover, they provide further insight into patient experience of body image changes and demonstrate important participant concerns after massive weight loss and body contouring surgery.

Future research in this area is needed. This could valuably take the form of recruiting patients from multiple surgery centres and embrace patients from varied ethnic groups, thus enhancing sample size and potential for generalisation. Moreover, studies could valuably include a prospective orientation. A longitudinal study might involve both qualitative and quantitative data collection, in order to both examine the experience of body change following body contouring surgery and assess perceptions of its significance via validated measurement tools. The qualitative element is essential in order to uncover the complex, dynamic, and constantly in “flux” nature of changing body image and general psychological well-being following body contouring surgery and to cast light on key factors and processes involved in adjustment including gender differences. Finally, greater consideration of culture, ethnicity and sexuality on perception of visible difference is also required in the context of this adjustment.

## 5. Conclusions

This research has provided new and interesting insights into changing body image following massive weight loss surgery, adding depth to existing, predominantly quantitative-based studies. It presents important evidence that sheds light on the huge body change that participants experienced, with the majority appearing to be in a continued state of flux in relation to their perceptions of body image and sense of well-being. The participants demonstrated strong determination throughout, leading on to bolstered resilience, positive lifestyle choices and a new sense of well-being. This trait enabled better adjustment to their changing bodies and promoted a greater sense of well-being. At the same time, some participants communicated high levels of internal and external shame, anxiety and low mood. This highlighted the importance of body acceptance in the present.

These findings, albeit from a small study and based on retrospective accounts, suggest important considerations to inform the provision of care. Nonetheless, the need for further research in a more diverse population sample would be valuable to establish whether interventions are needed for people experiencing difficulty adjusting to a changing body image after massive weight loss and body contouring surgery.
